# Insight Into Regulatory T Cells in Sepsis-Associated Encephalopathy

**DOI:** 10.3389/fneur.2022.830784

**Published:** 2022-03-16

**Authors:** Yu-lei Gao, Yan-cun Liu, Xiang Zhang, Song-tao Shou, Yan-fen Chai

**Affiliations:** ^1^Department of Emergency Medicine, Tianjin Medical University General Hospital, Tianjin, China; ^2^Department of Emergency Medicine, Rizhao People's Hospital of Shandong Province, Rizhao, China

**Keywords:** sepsis, sepsis-associated encephalopathy, Regulatory T cells, neuroinflammation, ischemic injury

## Abstract

Sepsis-associated encephalopathy (SAE) is a diffuse central nervous system (CNS) dysfunction during sepsis, and is associated with increased mortality and poor outcomes in septic patients. Despite the high incidence and clinical relevance, the exact mechanisms driving SAE pathogenesis are not yet fully understood, and no specific therapeutic strategies are available. Regulatory T cells (T_regs_) have a role in SAE pathogenesis, thought to be related with alleviation of sepsis-induced hyper-inflammation and immune responses, promotion of T helper (Th) 2 cells functional shift, neuroinflammation resolution, improvement of the blood-brain barrier (BBB) function, among others. Moreover, in a clinical point of view, these cells have the potential value of improving neurological and psychiatric/mental symptoms in SAE patients. This review aims to provide a general overview of SAE from its initial clinical presentation to long-term cognitive impairment and summarizes the main features of its pathogenesis. Additionally, a detailed overview on the main mechanisms by which T_regs_ may impact SAE pathogenesis is given. Finally, and considering that T_regs_ may be a novel target for immunomodulatory intervention in SAE, different therapeutic options, aiming to boost peripheral and brain infiltration of T_regs_, are discussed.

## Sepsis-Associated Encephalopathy: An Overview

Sepsis-associated encephalopathy (SAE) is a common, but poorly understood, diffuse central nervous system (CNS) dysfunction, that commonly appears in the setting of sepsis or systemic inflammatory response syndrome (SIRS) (1–3). Clinically, SAE is characterized by episodes of delirium, seizures, mild or deep unconsciousness, cognitive impairments, depression, decreased attention and motor coordination and social interaction problems (4, 5). This is the most common type of encephalopathy and one of the foremost causes of morbidity and mortality in patients in intensive-care units (ICU) worldwide. Moreover, SAE was shown to be associated with extensive in-hospital costs and prolonged hospitalization (6–8). Nevertheless, the epidemiological features and risk factors of SAE are not yet fully understood, and should be further explored in order to define suitable clinical interventions to reduce risk factors and thus, morbidity and mortality (9).

**Figure 1:**
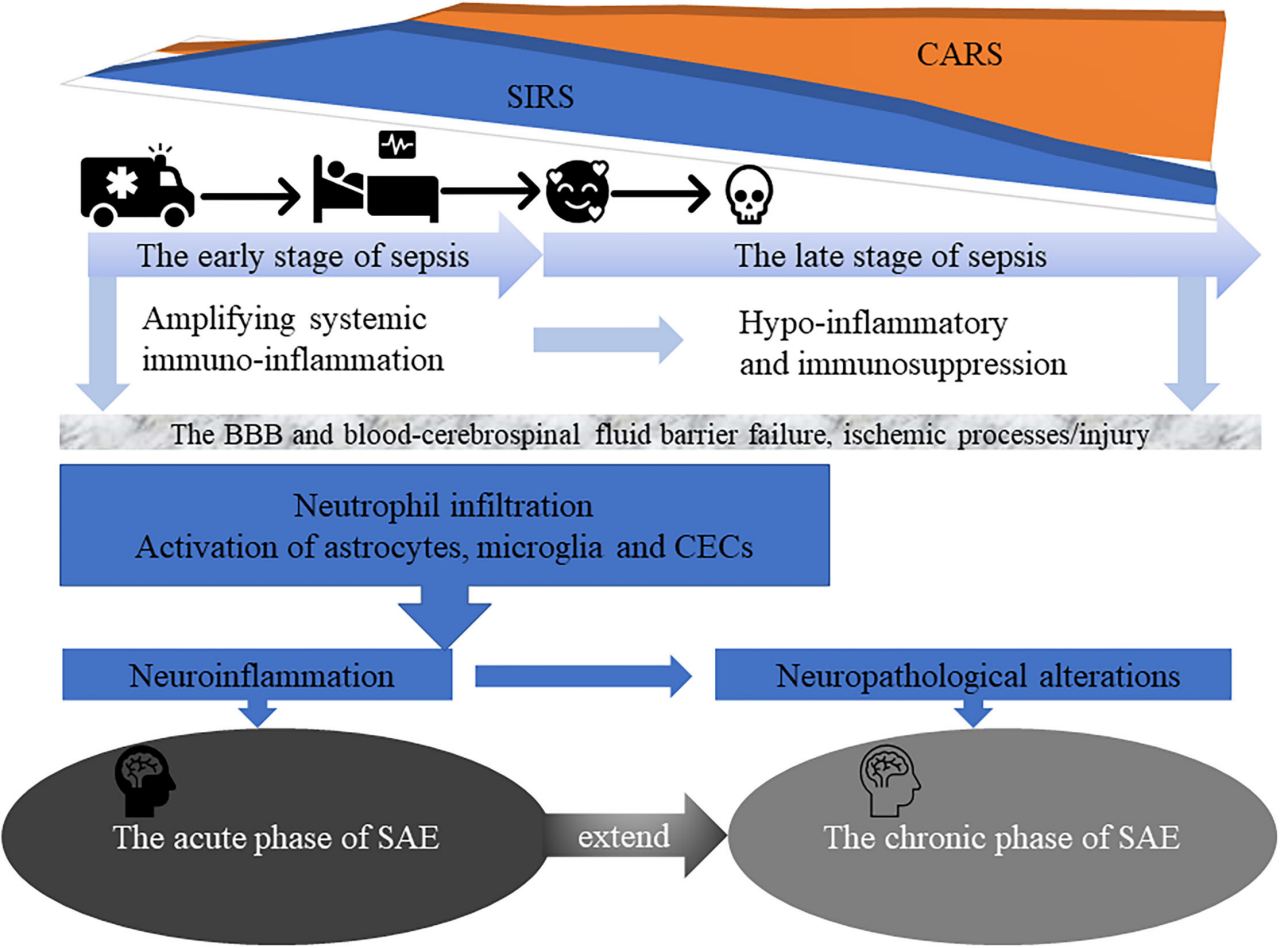
The early stage of sepsis is often accompanied by an acute phase of SAE characterized by delirium symptoms and acute changes in the patient's consciousness. In addition, in the late stage of sepsis, more than half of surviving patients gradually progress to the chronic phase of SAE suffering from severe and long-term cognitive deficits, and even depression, anxiety, post-traumatic stress disorder, and self-destructive tendencies, that affect their daily quality of life and place a significant burden on families and society.

The pathogenesis of SAE involves multiple intertwined factors, namely excessive production of pro-inflammatory cytokines, chemokine and acute phase proteins, including, but not restricted to interleukin (IL)-1β, IL-6, tumor necrosis factor (TNF)-α, Interferon (IFN)-gamma (γ), CXC-chemokine ligand 10 (CXCL10), C-reactive protein (CRP) and complement factors; endothelial cells activation/blood-brain barrier (BBB) collapse; glial cells activation; uncontrolled neuroinflammation; ischemic processes/injury; altered neurotransmission; mitochondrial dysfunction; apoptosis; and cognitive impairment (2–5, 7, 8, 10–13) (Figure 1). The role of each factor is patient-dependent and varies according to the clinical situation. Further studies need to be conducted to better understand the SAE pathophysiology and potentially develop new and more efficient therapeutic strategies.

From a clinical point a view, the disease course of SAE can be divided into acute and chronic phase (7, 9, 11, 14, 15). The acute phase of SAE is commonly characterized by delirium symptoms and acute changes in patient's consciousness (7, 9, 16, 17) and by the occurrence of different complex pathophysiological mechanisms, including uncontrolled neuroinflammation, as result of neutrophils, monocytes and resident glial cells activation and infiltration; long-term expression of multiple cytokines and chemokine genes, also called “cytokine storm” (18) and is thought to be the major cause of organ dysfunction and acute symptoms like fever, fatigue and anorexia; and enhanced T helper (Th)1 and Th17 cells activity (4) (Figure 1). These lead to dysfunction and massive apoptosis of brain cells, especially microglia, astrocytes, neurons and cerebral endothelial cells (CECs) (15, 19, 20). Indeed/accordingly, a study by Saito et al. (12) showed that in a cecal slurry (CS)-induced septic mouse model, neutrophils infiltration, an hallmark feature of SAE acute phase, favors CD4^+^ and CD8^+^ T cells accumulation, microglial activation and neuroinflammation (IL-1β and IL-6) in the cerebral cortex, BBB disruption, and increase anxiety-like symptoms. On the other hand, the number of astrocytes in the cerebral cortex and hippocampus was showed to gradually decrease. The complex pathophysiological mechanisms of SAE acute phase, may also have a long-term and chronic impact on the cognitive ability of sepsis survivors, and even in the development of dementia or neurodegenerative diseases (12, 21, 22). Sepsis may modulate neurodegenerative changes associated with Alzheimer's disease (AD), by favoring amyloid deposition and neuroinflammation in the brain (22, 23). Induction of polymicrobial sepsis by cecal ligation and puncture (CLP) significantly increased the formation of fibrillary amyloid plaques in the hippocampus of mouse models of AD-associated β -amyloidosis, enhanced intracranial plaque- related astrocytes activation and complement *C4b* expression, both of which regulate amyloid formation. Additional large-scale changes in the intestinal microbiota of mice were also associated with pro-amyloidosis and neuroinflammatory states (23).

As result of an increasing incidence and a decrease in mortality rates, sepsis survivors gradually progress to the chronic phase of SAE. This is often accompanied by severe and long-term cognitive deficits, at the level of memory, attention and verbal fluency; increased incidence of psychiatric disorders including, depression, anxiety, post-traumatic stress disorder and self-destructive tendencies; and inability to live independently. This strongly affects patient's quality of life and cause a significant burden to the families. Moreover, the need to keep a continuous monitoring on sepsis survivors, constitutes a major burden to the health care system, with high socio-economic costs (7, 9, 17, 24–26). Post mortem histology in humans who died from septic shock at the early stage of sepsis, indicate that the neuropathological alterations may be the starting point for permanent neurological damage in survivors, leading to long-term SAE - associated symptoms (4, 5, 16, 27–29). Early-onset neonatal sepsis can lead to an extremely complex set of events, like brain injury, and survivors remain vulnerable to both short- and long-term neurodevelopmental morbidity, due to long-term changes in cerebral blood flow, the release of neuroinflammatory proteins and altered metabolism (30).

## Sae and Regulatory T Cells

Regulatory T cells (T_regs_) are key players in immune regulation of both physiological and pathophysiological conditions, and are characterized by the expression of CD4, CD25 and forkhead box protein P3 (Foxp3). These cells have a negative immunomodulatory function, essential to maintain peripheral immune tolerance, prevent autoimmunity and limit chronic inflammatory diseases (31–34).

**Figure 2:**
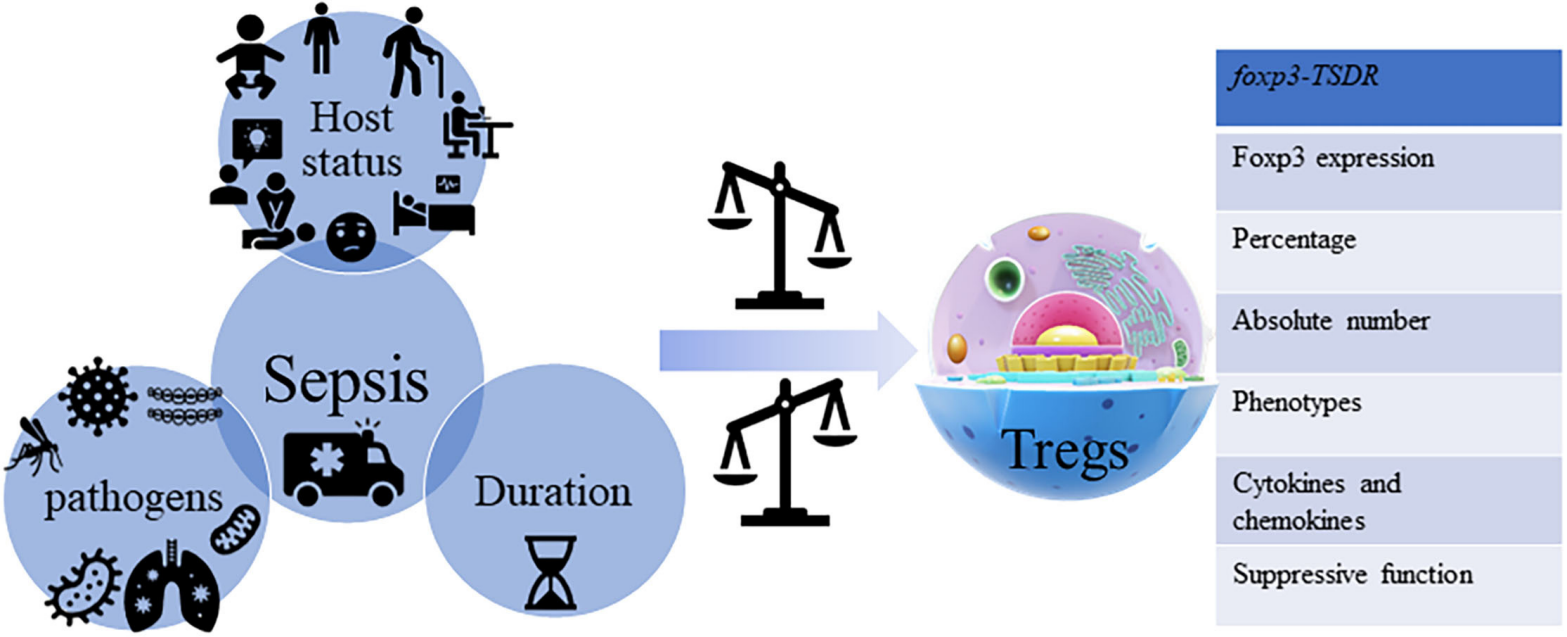
Sepsis and T_regs_. Sepsis is a kind of multi-dimensional heterogeneous syndrome, which is not only reflected in the host's demographics, chronic illness, comorbidities, laboratory abnormalities, infections position, patterns of organ dysfunction and severity of illness, and different types of the pathogen but also reflected in the protean host immune responses, where each is not identical. Sepsis influences the heterogeneous characteristics of T_regs_ from the aspects of percentage (CD4^+^CD25^+^/CD4^+^), absolute number, phenotypes [cytotoxic T lymphocyte antigen (CTLA)-4, CD25, PD-1, CD43, B- and T-lymphocyte attenuator (BTLA), neuropilin (Nrp)-1, G protein-coupled receptor (GPR) 174, lymphocyte activation gene (LAG)-3 and membrane-associated transforming growth factor-β (TGF-β^m+^), etc.], cytokines and chemokines [IL-10, TGF-β, IL-3, IL-35, and chemokine (C-X-C motif) ligand (CXCL)-4, etc.] secretion, and stability [Foxp3 expression, suppressive function, and methylation status of the *foxp3-T*_*regs*_*-specific demethylated region* (*TSDR*), etc.].

T_regs_ are one of the major subtypes of T cells, showing potent anti-inflammatory activity and mediating specialized functions in tissue remodeling (35). Several groups have been exploring the role of these cells on sepsis-induced immuno-inflammatory dysfunction (36–39) (Figure 2), however, their contribution in SAE pathogenesis, still remains unclear.

T_regs_ phenotype and function heterogeneity makes hard to define an universal comprehensive classification of the different T_regs_ subpopulations.

Based on their origin, T_regs_ can be divided into two subsets: thymus-derived T_regs_ (tT_regs_) and peripheral-derived T_regs_ (pT_regs_) (40–43). tT_regs_ (44) are characterized by their constitutive expression of Foxp3 and stable suppressive function, whereas pT_regs_ (45) exhibit unstable Foxp3 expression, unstable suppressive function and can differentiate into cells with an effector function. In a study conducted by Drechsler et al. it was demonstrated that pT_regs_, rather than tT_regs_, may play a role in improving septic survival (46). Increasing evidence, directly or indirectly, confirmed that pT_regs_ and brain-specific T_regs_ are involved in the acute and chronic phase of SAE by mitigating sepsis-induced hyper-inflammatory and immune responses, promoting Th2 functional shift, resolving neuroinflammation and improving the barrier function of the BBB endothelial cells, and even have the potential value of improving SAE-induced cognitive, behavioral and mental abnormalities (10, 12, 21, 47).

Other subsets of T_regs_ were defined based on cell phenotype and characteristic markers, secreted cytokines and chemokines and specific immune regulation functions (Table 1) (43, 48). These include Resting T_regs_ (rT_regs_), Activated T_regs_ (aT_regs_), Non-suppressive T_regs_ (non-T_regs_) and helper-like T_regs_ (Th-T_regs_), and three recently discovered Foxp3-T_regs_. Althoug there is still, to date, available studies exploring the impact of these subtypes in SAE, different studies demonstrated that distinct subsets with different functions could have a significant role on the control of the immune response and induction of peripheral tolerance (42).

**Table 1:** T_regs_ heterogeneity.

**T_**regs**_ subtype**	**Phenotype**	**Regulator**	**Cytokine/chemokine**	**Main function**
rT_regs_	CD25^++^CD45RA^+^Foxp3^low^CTLA-4^low^Ki-67^−^	Foxp3, Helios and *Foxp-3-TSDR*	None	Negative immune regulation, inhibiting excessive immune-inflammatory response, maintaining immune homeostasis
aT_regs_	CD25^+++^CD45RA^−^Foxp3^hi^ CTLA-4^hi^Ki-67^+^	Foxp3, Helios and *Foxp-3-TSDR*	IL-2, IFN-γ, IL-10 and TGF-β	
non-T_regs_	CD25^++^CD45RA^−^Foxp3^low^CTLA-4^int^	Foxp3, Helios and *Foxp-3-TSDR*	IL-2, IFN-γ and IL-17	
Th1-T_regs_	CXCR3^+^	Foxp3 and T-bet	IL-10, IFN-γ, CXCR3 and CCR5	Inhibits Th1, Th2, Th17 or Th22 cell responses and exerts pro-inflammatory effects
Th2-T_regs_	CCR4^+^ CCR6^−^ CXCR3^−^	Foxp3, GATA-3 and IRF-4	IL-10, IL-4, IL-13 and CCR4	
Th17-T_regs_	CXCR3^−^ CCR6^+^ CCR4^+^ CCR10^−^	Foxp3 and RORγt	IL-10, IL-17, CCR4 and CCR6	
Th22-T_regs_	CXCR3^−^ CCR6^+^ CCR4^+^ CCR10^+^	Foxp3	IL-10	
Th3	CD4^+^ LAP^+^ CD69^+^ CD25^low^ CTLA4^low^	Foxp3^−^	TGF-β1, IL-10 and IL-4	Promote Foxp3+T_regs_ differentiation and induce immune tolerance
T_regs1_	CD49b^+^ LAG3^+^ CD226^+^ CD25^low^ CTLA4^low^	Foxp3^−^, IL-10 and other cytokine	IL-10, TGF-β	
B-T_regs_	LAG3^+^ICOS^+^PD^1^+GITR^+^ OX40^+^CTLA4^+^	Foxp3^−^	IL-10	

T_regs_ may impact the pathophysiological mechanism of sepsis by acting on the innate and the adaptive immune system, weakening immune function, causing immunoparalysis, and eventually leading to multiple organ dysfunction syndromes and death in sepsis (37, 49). T_regs_ seem to be key players in the development of sepsis, as well as the hotspot strategies in immunotherapy and immune checkpoints of sepsis and sepsis-associated complications. However, the dual functions of T_regs_ in infections may provide beneficial or harmful effects even though the number of CD3^+^ CD4^+^ CD25^hi^ CD127^lo^T_regs_ in the early stage of sepsis (within 3 days) is not associated with the outcomes (36–39, 50–59).

The brain-specific T_regs_ of normal rat are memory T cells that account for a larger proportion of cerebral T cells than in the periphery, and, as in the periphery, also play a key role in immune tolerance. The brain-specific T_regs_ have immunosuppressive effects, including control glial cells activation, with high expression of anti-inflammatory cytokines (IL-10 and IL-35) and immunosuppressive molecules (Foxp3, CTLA-4, CD39, and CD73) (60). Complex systems between the nervous system and the immune system have evolved to mitigate the effects and facilitate recovery from brain injuries caused by diseases such as traumatic brain injury (TBI), ischemic stroke, AD and other degenerative neuropathies, as well as different kind of encephalopathies, including SAE, etc. (16, 17, 61, 62). T_regs_ infiltrated the brain 1–5 weeks after experimental ischemic stroke in mice and had potent immunomodulatory effects on other immune cells, including monocytes. The interaction between infiltrated T_regs_ and microglia via T_regs_-derived osteopontin in the brain is crucial in behavioral recovery, brain repair, and long-term outcomes (62).

## The Mechanisms of T_regs_ in SAE

**Figure 3:**
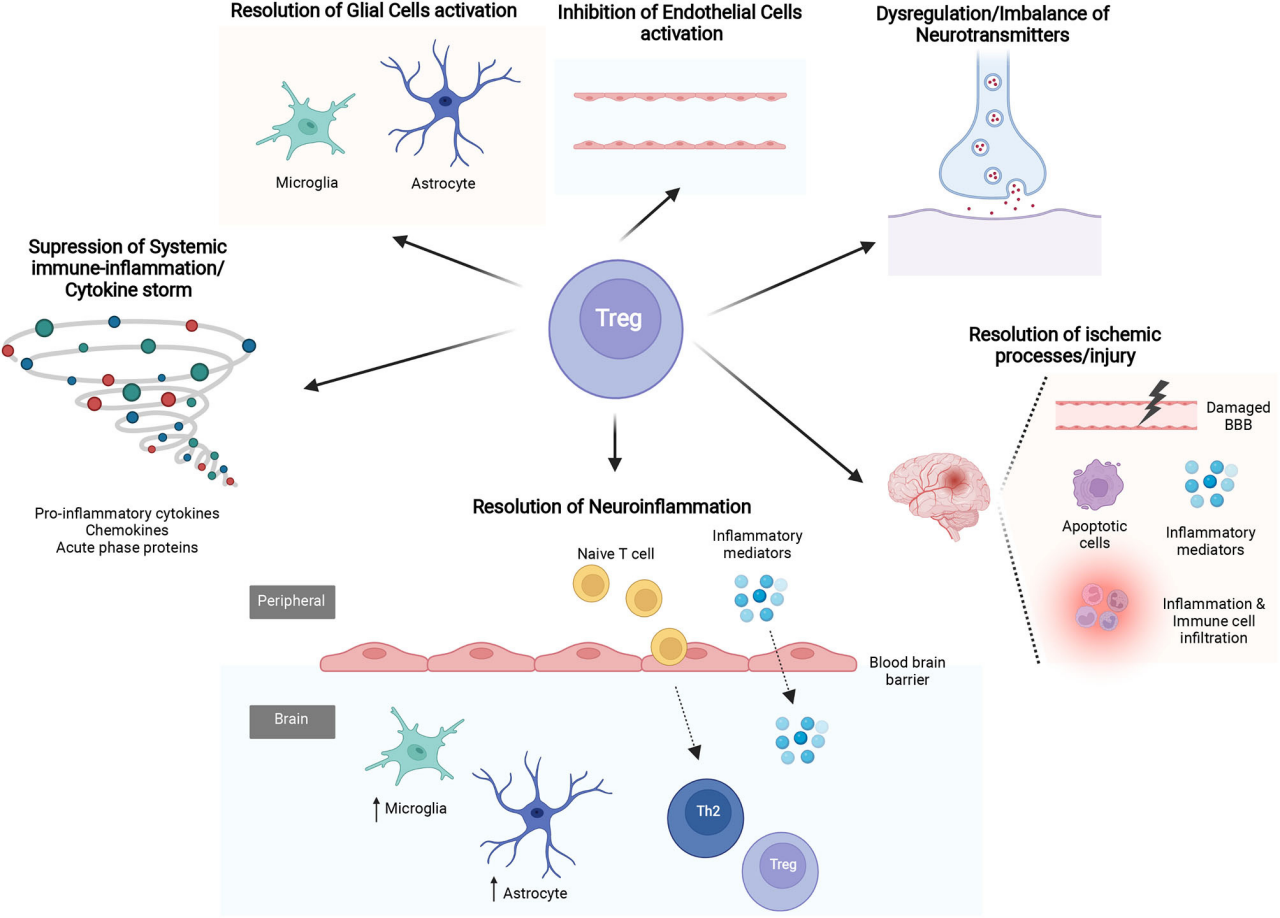
The mechanisms of Regulatory T cells (T_regs_) in sepsis-associated encephalopathy (SAE). Several studies evidence the mechanisms by which T_regs_ may affect different processes know to be related with SAE pathophysiology. The latter include suppression of systemic immune-inflammation/cytokine storm, resolution of excessive neuroinflammation and ischemic processes/injury, regulate the imbalance of neurotransmitters and regulation of glial cells and cerebral endothelial cells (CECs) activity.

SAE is partly mediated by the dysregulated immune response to sepsis, nevertheless, the pathophysiology underlying it, is still largely unknown. With this in mind and extrapolating the knowledge that T_regs_ may positively impact different processes, in this section, we give an overview of the main mechanisms by which T_regs_ may regulate SAE pathogenesis (Figure 3; Table 2).

**Table 2:** Mechanisms of T_regs_ in SAE.

**Pathogenesis mechanism**	**Main goal of the study**	**Disease model**	**Main outcome**	**Relevance in SAE**	**References**
Imbalance/dysregulation of neurotransmitters	Role of acethylcoline (Ach) in the inflammatory response in survivors of sepsis, through activation or suppression of cholinergic transmission	Mice model of experimental sepsis induced by cecal-ligation and puncture (CLP)	Acethylcoline reduces inflammation, in the brain and spleen, by promoting the proliferation of T_regs_ and decreasing pro-inflammatory cytokines expression	Cholinergic anti-inflammatory pathway is the main pathway dysregulated in SAE and controls the inflammatory response and non-reflexive consciousness	(63)
Ischemic processes/injury	Therapeutic impact of allogenic adipose-derived exosomes (ADMSC) on the early targeting of inflammatory signaling and on the protection of the brain from sepsis syndrome-induced injury	Rat model of sepsis syndrome (SS)-induced by CLP (CLP)	Cell-derived exosomes (AMSC^EXO^) markedly suppress the systemic immune-inflammatory responses and protect the brain against SS-induced injury	Mesenchymal stem cell (MSC)-derived exosomes regulate the inflammatory-oxidative signaling axis and protect the organs from sepsis or ischemic-reperfusion damage	(10)
Cerebral endothelial cells activation	Explore the function of tissue-non-specific alkaline phosphatase (TNAP) at the brain-immune axis in experimental sepsis	Sublethal mice model of experimental sepsis induced by CLP	TNAP protects against the loss of BBB permeability and improves survival, clinical scores and behavioral outcomes associated with early sepsis	Alkaline phosphatases have a protective role at endothelial barriers and may shape the dynamic interactions within the brain-immune axis	(47)
Systemic immune-inflammation/cytokine storm; neuroinflammation; ischemic processes/injury; glial cells activation	Understand how immune cells, and more specifically T cells, influence SAE pathogenesis	Mice model of experimental sepsis induced by CLP	T_regs_ and Th2 infiltration resolves neuroinflammation and contributes for SAE attenuation and SAE-induced mental disorder	T cells infiltrating the brain during sepsis have an impact on the attenuation of specific SAE pathogenesis mechanisms and on the development and recovery of mental impairment in septic survivors	(12)

*Summary of the studies available, to date, evidencing the potential mechanisms by which T_regs_ may affect SAE pathogenesis*.

### Suppression of Sepsis-Induced Systemic Immune-Inflammation/Cytokine Storm

The systemic immune-inflammation/cytokine storm, which constitutes an exaggerated host immune response associated with excessive production of pro-inflammatory cytokines, chemokines and acute phase proteins, is a hallmark feature in the acute phase of sepsis (18, 64–71). This is one of the main causes of death and ICU acquired complications, including SIRS and compensatory anti-inflammatory response syndrome (CARS), and is defined by the host status (57, 72–76), pathogens (77, 78) and the time span of sepsis (71, 79–84). T_regs_ have been shown to have a role on the inhibition of sepsis-induced amplifying systemic immuno-inflammation/cytokine storm and on the protection of organs in the early stage of sepsis (53, 85, 86). More specifically, a study conducted by Tatura et al. (51), evaluated the role of T_regs_ in the early and late stage of sepsis, using a DEREG (Depletion of REGulatory T cells) mouse model, in which sepsis was induced by CLP and subsequent *Pseudomonas aeruginosa* pulmonary infection. DEREG mouse exhibit high disease scores, mortality, and IL-6 expression in the early stage of sepsis. This study thus, corroborates the hypothesis that T_regs_ can be explored to limit sepsis-induced amplifying systemic immuno-inflammation/cytokine storm and accelerate a positive outcome in the early stage of sepsis. The suppressive role of T_regs_ in the sepsis-induced immune-inflammation/cytokine storm was also demonstrated in a study using G protein-coupled receptor 174 (GPR174)-deficient mice (53). In an initial phase of sepsis, this mouse model present a higher expression of IL-10 and CTLA-4 in Tr_egs_, reduced tissue damage and promotion of macrophage polarization to an M2 phenotype induced by sepsis via T_regs_. Moreover, an enhanced supressive function of T_regs_ on IL-6 and TNF-a secretion was also showed. Overall, this study corrobarates the prominent role of T_regs_ in autoimmune tolerance and in restraining the exagerated immune activation in a sepsis context.

### Resolution of Neuroinflammation

Neuroinflammation is a critical mechanism in the pathogenesis of SAE, characterized by microglia activation, astrogliosis and infiltration of peripheral inflammatory mediators and immune cells (5, 20). Therefore, studies exploring mechanisms that allow the amelioration of this process, may be of great interest in the context of SAE. With this in mind, and considering the evidences suggesting that T_regs_ may contribute to recovery from SAE and mental impairment, Saito and colleagues (12), hypothesize that T cells, and more specifically T_regs_ infiltrating the brain, may contribute to the attenuation of SAE and the alleviation of SAE-induced anxiety-like behavior. For that, they used a cecal slurry-induced septic mouse model. A batch of behavioral tests (open-field test, marble burial-test and forced swimming test) was conducted to assess the anxiety-like behavior/state in the septic mouse model. In addition, neuroinflammation and T cell infiltration were also examined in the cerebral cortex. They reported that the infiltration of T cells, and more specifically T_regs_ and Th2 cells, in the chronic phase of SAE, reduced SAE-induced mental impairment by alleviating neuroinflammation (12, 61). Interestingly, they also showed that the administration of Fingolimod (FTY720), a sphingosine 1-phosphate antagonist that can inhibit lymphocyte escape from lymph nodes, delayed resolution of neuroinflammation and remission of depression in septic mice. This finding was showed to be associated with FTY720's ability to decrease the infiltration of T_regs_ and Th2 cells and increase Th17 cells in the brain (12). Overall, these findings suggest that infiltrated T_regs_ and Th2 cells have a promising role in the resolution of neuroinflammation and associated anxious/depressive behavior, which can be of great interest for the investigation of new potential therapeutic targets.

### Resolution of Ischemic Processes/Injury

Ischemic processes/injury have been, together with neuroinflammation, identified as playing a critical role in SAE pathogenesis. These mechanisms are directly associated with BBB disruption, brain edema, monocytes and macrophages infiltration, astrogliosis and brain cells apoptosis (11, 14, 15). The aforementioned issues raise the hypothesis that early targeting of the inflammatory signaling may have a potential therapeutic role in the protection against the acute phase of SAE. In this regard, Chang and colleagues, showed that the use of allogenic adipose-derived mesenchymal stem cell-derived exosomes (AMSC^EXO^) markedly protect the brain against sepsis-induced ischemic injury in rat with sepsis syndrome (10).

Reductions in T lymphocytes, especially the cerebral infiltration of T_regs_, is consistent with increased infiltration of natural immune cells, including neutrophils and inflammatory macrophages, and hypoxic-ischemic injury-induced brain injury, including loss of gray and white matter structures (12, 87). C-C chemokine receptors (CCR), such as CCR2, CCR5, and CCR7, are critical molecules for T_regs_-mediated BBB protection and potential targets for optimizing T_regs_ therapy for the treatment of neurological diseases such as ischemic stroke and AD (88–90). CCR5 is critical for T_regs_ docking at the injured vascular wall, where they interact with blood-borne neutrophil/macrophage. Donor T_regs_ were deficient in CCR5 and lost their early protective effect against cerebral ischemic processes/injury (88).

### Imbalance/Dysregulation of Neurotransmitters

Neurotransmitters are likely to be involved in the development and maintenance of SAE. Indeed, different studies have highlighted cholinergic pathway as the main pathway being dysregulated in an SAE context. However, complementary studies showed that other pathways, including gamma-aminobutyric acid, norepinephrine, serotonin and dopamine, seem also to be compromised (91, 92).

Neurotransmitters are traditionally referred as chemical messengers that trigger or inhibit the functions of neurons. However, they can also bind neurotransmitter receptors in T cells and directly activate or suppress their function. Additionally, T cells produce endogenous neurotransmitters and can be regulated by them in an autocrine/paracrine manner (33, 93). More specifically, T_regs_ were shown to selectively express tyrosine hydroxylase and contain endogenous catecholamines, which function in the autocrine/paracrine inhibitory loop (94). Dopamine down-regulates T_regs_ activity through the extracellular signal-regulated kinase pathway (95). Acetylcholine reduces inflammation by promoting the proliferation of T_regs_ in the hippocampus and spleen (63). The α7 nicotinic acetylcholine receptor (α7nAChR), a ligand-gated ion channel, plays an important role in inflammatory responses and is also expressed on the surface of T_regs_. Activation of α7nAChR could increase the number and activation of T_regs_ through the α7nAChR/p-Erk/Foxp3 signaling pathway, and play an anti- neuroinflammatory role in the process of 6-hydroxydopamine (6-OHDA)-induced injury (96). Nitric oxide (NO) production seems to be important for regulating metabolic homeostasis and immune response during sepsis. Arginine (Arg)-induced NO inhibition disrupted the beneficial effect of Th1/Th2 and Th17/T_regs_ distribution, suggesting that the Arg-NO pathway may partly regulate Th/T_regs_ homeostasis during sepsis (97).

### Resolution of Glial Cells Activation

The activation of microglia, the brain's macrophages, is involved in the progression of SAE by disrupting the BBB function, increasing acute hippocampal neuroinflammation, and enhancing the release of reactive oxygen species (ROS) and consequently, mitochondrial injury (19, 47, 98, 99). Interestingly, while on the seek to better understand SAE pathogenesis and found novel molecular treatments and therapeutic strategies, a recent study explored the protective role of stanniocalcin-1 (STC-1), a glycoprotein-secreted hormone, present in brain, as well as other tissues, in a rat model of sepsis (99). STC-1 decreased microglia-induced acute hippocampal inflammation and oxidative stress and increased the activity of mitochondrial respiratory chain and creatine kinase, after 24 h, thus conferring protection against SAE. Overall, this work demonstrates that strategies promoting neuroprotection by inhibiting the inflammatory response in microglia and protecting against sepsis-associated encephalopathy, have great potential on the treatment of SAE.

The use of cell therapies to reduce microglia-mediated inflammatory response have been explored and can be of great interest as a therapeutic strategy for SAE. More specifically, the use of human mesenchymal stem cells (MSCs) in combination with human cord blood T_regs_, favored a reduction of microglial proliferation and activation after TBI and altered the systemic immuno-inflammation (100). The brain-specific T_regs_ inhibit microglia/macrophage-mediated inflammation via IL-10 and TGF-β, while the homeostatic astrocytes maintain the heterogeneous characteristics of brain-specific T_regs_, *via* IL-2/STAT5, IL-33, CCL1-CCR8, CCL20-CCR6 signaling pathway, as helper cells (60, 101).

Astrocyte activation was detected in brain tissues 4 h after sepsis, peaking at 24 h, and thus, favoring abnormal responses, including the decrease of mitochondria biogenesis and the secretion of inflammatory cytokines through nuclear factor (NF)-κB and other signaling pathways in the astrocytes of the cerebral cortex. Consequently, inflammatory brain injury, refractory neuroinflammation and cognitive impairment are observed (98, 102–104). In addition, extensive structural changes of astrocytes, such as structural remodeling and loss of endfeet, are responsible for BBB collapse (105). Functional T_regs_ play a critical role in inhibiting astrocyte activation through the production of amphiregulin (AREG), which binds epidermal growth factor receptor (EGFR) on astrocytes and inhibits IL-6 production from astrocytes STAT3 pathway (106). In FTY720-treated septic mice, the number of astrocytes in the cerebral cortex is still reduced at day 30, suggesting a role of infiltrated Tregs on the number and activity of astrocyte in sepsis (12).

### Inhibition of Cerebral Endothelial Cells Activation

Abnormal function and cell death of CECs, as result of neuroinflammation, showed great potential to accelerate SAE in the early stage of sepsis. This happens as result of the formation of a direct link between neurovascular inflammation and brain injury through the P2RX7 pathway, increased vascular inflammation and infiltration of inflammatory cells (CD11b/CD18-expressing leukocytes), abnormal migration of microglia and excess of ROS and NO (47, 98, 103, 107). Gene expression profiles of cerebral vessels isolated from the brains of peripheral LPS-treated mice revealed that, cerebral vessels respond to acute systemic inflammation within minutes by up-regulating the expression of immediate early response genes, followed by the activation of NF-κB pathway. Activation of CECs is the earliest event at the onset of SAE and is the most likely the primordial source of neuroinflammation favoring glial activation. Subsequently, apoptotic signals are activated in CECs, which are thought to result in the BBB disruption and allow leakage of peripheral cytokines into the CNS, exacerbating gliosis and leading to a malignant neuroinflammatory cascade (20). In the setting of cholestatic liver injury, T_regs_ have the ability to modulate the development of sickness behavior (social investigative behavior and immobility), primarily by inhibiting circulating monocytes and hepatic IL-6 production, and subsequently by circulating IL-6/STAT3 signaling that acts at the level of hippocampal CECs (108). The expression of CCL5, a CCR5 ligand, is significantly increased on the injured CECs after cerebral ischemia and is accompanied by upregulation of CCR5 expression on circulating T_regs_. In the co-culture of T_regs_-CECs, when T_regs_ are exposed to ischemia-injured CECs, CCR5 expression is induced. In addition, CCR5 induction on T_regs_ enhanced the expression of inhibitory molecular PD-1L, thereby inhibiting neutrophil-derived matrix metallopeptidase 9 (88).

## T_regs_-Related Therapeutic Potential in SAE

The prominent role of T_regs_ in the regulation of SAE pathogenesis, makes these cells highly attractive for the development of novel therapeutic strategies aiming to modulate the immune response, improve patient's clinical outcome and reduce the associated mortality. However, there is still a lack of knowledge that limits the application of these cells in a SAE therapeutic context. With this in mind, and considering the reduced number of studies available to date exploring T_regs_ therapeutic potential in SAE, in this section we give an overview on potential ways in which T_regs_ can be therapeutically used.

### Inducing the Accumulation of T_regs_ in the Brain

For decades, dementia has been characterized by a buildup of waste in the brain and mild inflammation, and was shown to be influenced by the immune system (89). In a rat model of sepsis, exposure to LPS for 7 days leads to deposition of amyloid-β plaques and phosphorylated tauopathy in the hippocampus (23, 109). In acute pathologies, such as TBI and ischemic stroke, CD4^+^ T cells and Foxp3^+^ T_regs_ are recruited into the injured tissue to promote its repair (61, 62, 88, 110). Blocking the programmed cell death Ligand 1 (PD-L1) pathway for 12 days in a DM-hTAU transgenic mice model (a mouse model of tauopathy), favored an increased accumulation of Foxp3^+^T_regs_ in the brain via the CCR 2/CCL 2 axis. Simultaneously, an improvement of the cognitive behavior, disease pathology (mainly phosphorylated tauopathy deposition in the hippocampus), neuronal survival and hippocampal inflammation, was also observed (89). Nevertheless, the increased accumulation of Foxp3^+^T_regs_ in the brain can also have detrimental effects. With this in mind, it was shown that anti-CD25 antibodies can be used to mitigate the accumulation of Foxp3^+^T_regs_, which in turn can alleviate aging and neurodegenerative diseases caused by CCR7-dependent egress of immune cells (90). Increasing the number of T_regs_, by delivering the IL-2 antibody complex after ischemic stroke, improves white matter integrity and rescue neurological functions over the long term. These findings suggest that T_regs_ are the neurorestorative target for stroke recovery (62). Gold nanoparticles, such as 20 nm citrate-covered gold nanoparticles (cit-AuNP), have been demonstrated to have important anti-inflammatory properties in the brain of sepsis and are promising as adjunctive agents in sepsis treatment with antibiotics to avoid SAE (111). Hyperforin-loaded gold nanoparticles, gold nanorods/compounds and anti-inflammatory nanoparticles have the ability to improve symptoms by inhibiting the differentiation of Th1 and Th17, while promoting the accumulation of T_regs_ and Th2 in the treatment of EAE, cancer, and muscular dystrophy (112–114). Therefore, gold nanoparticles can have therapeutic interest to promote T_regs_ accumulation in the context of SAE.

### Establishing the Brain-Specific T_regs_

The primary role of T_regs_ in lymphoid tissues is defense, while the primary role of tissue-specific T_regs_ located in non-lymphoid parenchymal tissues (e.g., skin, muscle, gastrointestinal tract, lung, adipose tissue, central nervous system, etc.) is to maintain homeostasis (12, 35, 115). Many of these tissue-specific T_regs_ functions, go beyond our initial understanding of T_regs_ as immuno-inflammation-specific inhibitors (84, 86), whereas a large number of previous intervention and observational studies on sepsis have focused on the functions and characteristics of T_regs_ in the peripheral circulation and spleen (51–53, 75, 77). The timing and location of tissue-specific T_regs_-mediated immune homeostasis and regulation, remain undefined. In the EAE model, at the peak of EAE-related symptoms, infiltrated brain-specific T_regs_ in the brain tissue, such as Blimp1-expressing follicular T_regs_ and TNF receptor (TNFR2) 2 -expressing T_regs_, are critical in suppressing EAE, maintaining the continuous expression of CTLA-4 and Blimp-1, and allowing active suppression of pathogenic T cells in the brain, but have no effect on T cell in peripheral lymphoid tissue (116, 117). Selective depletion of the brain-specific T_regs_ decreases oligodendrogenesis, white matter repair and functional recovery, after experimental ischemic stroke. The beneficial effects of T_regs_ on white matter regeneration were mitigated by microglia depletion. T_regs_-derived osteopontin acts on microglia via integrin receptors to enhance microglial reparative activity (62).

### Regulating the Neuroendocrine-Immune Network

CNS and endocrine system dysfunction, as well as peripheral immuno-inflammatory system collapse, are mutually causal in sepsis. In addition, the CNS through the hypothalamic-pituitary-adrenal (HPA) axis, gut-brain axis, sympathetic and parasympathetic nervous system, function as a transportation hub (8, 13). The immuno-inflammatory signals affect different regions of the brain, mainly through humoral and neural pathways, which mostly involve damage of the BBB and activation of vagal afferent fibers, respectively (4, 5, 8, 59). At the same time, CNS dysfunction may be an important cause of neuroendocrine-immune network breakdown, as well as a potential therapeutic target (such as cholinergic anti-inflammatory pathway, humoral pathway mediated by vasopressin, and reconstruction of the HPA axis) for sepsis-induced immunosuppression or endocrine dysfunction (19, 63, 118). The dysregulation of cholinergic and inflammatory systems are the main pathophysiologic mechanisms regulating other brain injuries (such as hepatic encephalopathy and ischemic encephalopathy). The cholinergic anti-inflammatory pathways, control inflammatory responses and non-reflexive consciousness through two-way communication between the brain and the immune system. In this regard the activity of brain acetylcholinesterase is especially important, which is of great benefit to brain injury and is a promising neuroprotective therapy (8, 19, 63, 119). The cholinergic transmission, stimulated by administration of Donepezil, reduces the inflammatory response, 15 days after CLP, by promoting the proliferation of T_regs_ in the hippocampus and spleen, whereas the homozygous mutant vesicular acetylcholine transporter-knockdown (VAChT-KD) reduced the number of T_regs_ in the hippocampus and increased inflammation (63).

### Regulating the Gut-Brain Axis

The gut-brain axis is a bidirectional signaling network of neurons, hormones, immune cells, and microbial molecules that communicate in different ways, namely through the vagus nerve and the enteric nervous system, which can communicate to the brain using gut metabolites (120). The enteric nervous system (ENS) is the largest nerve organ outside the brain and operates autonomously, responding and adapting to local challenges. The immune system and ENS monitor the boundaries between commensal and pathogenic microorganisms in the colon, and FoxP3^+^ T_regs_ functionally interact with ENS (121, 122). In the colonic lamina propria, microbial-responsive RORγ^+^ and Helios^+^ subsets of T_regs_, closely respond to nitrogen energy and peptide nerve fibers. Intestinal neurons inhibit pT_regs_ differentiation and regulate RORγ^+^ T_regs_ ratio by secreting IL-6. T_regs_ and ENS constitute a regulatory circuit in which microbial signals regulate neuronal density and activation, thereby regulating T_regs_ generation and immune tolerance in the gut (120). The immune signals following sepsis or altered gut composition can cause the brain to respond to a perceived threat of infection and trigger an inflammatory response (123). Patients with sepsis, experience ectopic intestinal flora caused by acute stress, while survivors also experience chronic stress-induced depression and anxiety initiation mediated by gut-brain-axis immunity. The gut-brain-microbe axis is a promising therapeutic target for stress-induced behavioral injury, as it modulates both the peripheral and cerebral immune landscapes (121–123). The combination of probiotics and prebiotics promotes behavioral resilience to chronic stress by normalizing the gut microbiome and promoting T_regs_ expansion, an impact reflecting behavioral responses better than neuroinflammation in limbic regions of brain. The ratio of ileal T_regs_/Th17 is associated with the production of hippocampal chemokines and IL-1β in the prefrontal cortex (124). Traditional Chinese Medicine, such as resveratrol, may inhibit neuroinflammation by regulating microbiota-gut-brain axis mediated Th17/T_regs_ and Th1/Th2 polarity shift (125).

Commensal gut bacteria impact the host immune homeostasis and can affect disease processes in the brain, through the microbiota-gut-brain axis (120). IL-17^+^ γδ T cells can be transported from the gut to the brain, especially to the leptomeninges, and enhance ischemic injury through the secretion of IL-17. This results in increased levels of chemokines in the brain parenchyma and subsequent infiltration of cytotoxic immune cells, including neutrophils. Amoxicillin/clavulanate-induced alterations in the intestinal flora or fecal transplants, favors a reduction of the ischemic processes/injury. This happens as result of an increase of intestinal T_regs_ and a reduction of IL-17^+^ γδ T cells in the small intestine. Indeed, T_regs_ induction could be the main mechanism leading to IL-17^+^ γδ T cells inhibition (126). The farnesoid X receptor (FXR) signaling system closely links gastrointestinal and non-gastrointestinal tissues, especially the gut-brain-immune axis. Regulation of FXR promotes the production of T_regs_ and protects the brain against ischemic processes/injury (122).

Overall, the studies described, showed us the importance of further studies to better understand the effects of the intestinal microbiome on the induction and function of T_regs_, and more specifically, study the crosstalk between the enteric nervous system and T_regs_ in the context of SAE.

### Adoptive Cell Transfer Therapies With T_regs_

Adoptive cell transfer (ACT) therapies with T_regs_ have been used in the clinic to manage different immune-related diseases, especially autoimmune diseases and tumors, and animal studies have shown great promise against neurological disorders such as AD, Parkinson's disease, and ischemic stroke (127, 128). In adult offspring with maternal-immune activation (MIA), ACT therapies using activated T_regs_ (CD4^+^CD25^+^Foxp3^+^) largely restores MIA-induced pro-inflammatory profile and reverses behavioral abnormalities. Moreover, through the use of pathogen-activated maternal T_regs_ (pathogen-specific maternal T_regs_), ACT therapies favor an increase of anti-inflammatory IL-10 level by ~13-fold in the hippocampus (128). T_regs_ depletion exacerbates inflammation and increases cerebral infarction after ischemic stroke, while the increase in T_regs_ levels after stroke can prevent further exacerbation of stroke (62, 88). In the acute phase of ischemic stroke, T_regs_-based ACT therapies decreases the infiltration of peripheral leukocytes into the infarct area, inhibits cerebral inflammation, and improve the integrity of the damaged BBB (88). In a model of neuromyelitis optica spectrum disorder (NMOSD), T_regs_-based ACT therapy, through intraperitoneal injection, immediately after induction of NMOSD can have a protective effect on the brain injury by reducing the systemic inflammatory response in the acute phase of the disorder. At the same time, it can reduce the infiltration of macrophages, neutrophils, and T cells, and decrease the level of chemokines and pro-inflammatory cytokines in brain tissue (129).

### Stem Cell-Derived Small Extracellular Vesicles

Several studies have shown that stem cell-derived small extracellular vesicles have angiogenesis, immunomodulatory, anti-inflammatory, neurological and paracrine effects. These may have potential therapeutic value for SAE, as they may act by regulating sepsis-induced systemic immuno-inflammatory response, dysfunction of the neuroendocrine network, diffuse neuroinflammation, ischemia and imbalance of neurotransmitters (10, 81, 98, 110). The therapeutic effect of human adult stem cells in protecting against the early stage of severe sepsis is partly related to the induction of IL-10-secreting T_regs_, thereby reducing inflammatory cells infiltration in different target organs and down-regulating the production of various inflammatory mediators (85). In this regard, Chang et al. showed that use of AMSC^EXO^can effectively inhibit systemic inflammation induced by sepsis in rats, by increasing the number of circulating and splenic T_regs_, while decreases the number of CD3^+^/CD4^+^ and CD3^+^/CD8^+^ cells, thus having a significant protective effect on brain injury induced by sepsis (10). Embryonic stem cell-derived small extracellular vesicles (ESC-sEVs) protect ischemic stroke and modulate post-stroke immune responses by regulating T_regs_ through the TGF-β/Smads signaling pathway, and the depletion of T_regs_ almost completely abrogates the protective effects of ESC-sEVs (110).

## Conclusion

SAE is a multifaceted disorder with significant impact on patient's morbidity and mortality, and whose pathogenesis is complex and multifactorial and not yet completely understood. T_regs_ have been shown to play a critical role in SAE and thus are gaining increased attention as subject of research in its pathogenesis and treatment. However, the heterogeneity and plasticity of these cells, together with the complexity of the immune system, constitute a significant challenge when envisaging their therapeutic use. With this in mind, an increased understating of the alterations in human T_regs_ function in patients during SAE, together with animal studies addressing mechanisms underlying this phenomenon, will be greatly helpful to clarify the potential role of T_regs_ in patient's outcome after SAE.

## Author Contributions

Y-lG, Y-cL, and Y-fC generated the concept for this review and performed the literature search. Y-lG, Y-cL, XZ, and S-tS revised the draft. S-tS and Y-fC guided the study. All authors have read and approved the final manuscript.

## Funding

This work was supported by the National Natural Science Foundation of China (Nos. 81871593 and 81701931), and the National Natural Science Foundation of Tianjin (No. 18JCQNJC10500).

## Conflict of Interest

The authors declare that the research was conducted in the absence of any commercial or financial relationships that could be construed as a potential conflict of interest.

## Publisher's Note

All claims expressed in this article are solely those of the authors and do not necessarily represent those of their affiliated organizations, or those of the publisher, the editors and the reviewers. Any product that may be evaluated in this article, or claim that may be made by its manufacturer, is not guaranteed or endorsed by the publisher.
